# Interplay Between the Gut-Brain Axis, Obesity and Cognitive Function

**DOI:** 10.3389/fnins.2018.00155

**Published:** 2018-03-16

**Authors:** Ana Agustí, Maria P. García-Pardo, Inmaculada López-Almela, Isabel Campillo, Michael Maes, Marina Romaní-Pérez, Yolanda Sanz

**Affiliations:** ^1^Microbial Ecology and Nutrition Research Unit, Institute of Agrochemistry and Food Technology, National Research Council (IATA-CSIC), Valencia, Spain; ^2^IMPACT Strategic Research Centre, School of Medicine, Deakin University, Geelong, VIC, Australia

**Keywords:** microbiota, cognition, mood, behavior, obesity

## Abstract

Obesity continues to be one of the major public health problems due to its high prevalence and co-morbidities. Common co-morbidities not only include cardiometabolic disorders but also mood and cognitive disorders. Obese subjects often show deficits in memory, learning and executive functions compared to normal weight subjects. Epidemiological studies also indicate that obesity is associated with a higher risk of developing depression and anxiety, and *vice versa*. These associations between pathologies that presumably have different etiologies suggest shared pathological mechanisms. Gut microbiota is a mediating factor between the environmental pressures (e.g., diet, lifestyle) and host physiology, and its alteration could partly explain the cross-link between those pathologies. Westernized dietary patterns are known to be a major cause of the obesity epidemic, which also promotes a dysbiotic drift in the gut microbiota; this, in turn, seems to contribute to obesity-related complications. Experimental studies in animal models and, to a lesser extent, in humans suggest that the obesity-associated microbiota may contribute to the endocrine, neurochemical and inflammatory alterations underlying obesity and its comorbidities. These include dysregulation of the HPA-axis with overproduction of glucocorticoids, alterations in levels of neuroactive metabolites (e.g., neurotransmitters, short-chain fatty acids) and activation of a pro-inflammatory milieu that can cause neuro-inflammation. This review updates current knowledge about the role and mode of action of the gut microbiota in the cross-link between energy metabolism, mood and cognitive function.

## Introduction

The microorganisms inhabiting the mammalian's intestinal tract (collectively termed microbiota) include bacteria, viruses, protozoa, archaea, and fungi, with bacteria representing a majority (Gill et al., 2006; Xu et al., 2007). This is an extremely complex ecosystem; in particular, the human adult gut microbiota is estimated to comprise over 1000 different bacterial species with more than 7000 strains (Ley et al., 2006a; Qin et al., 2010). The collective genome of the microbiota (termed microbiome) exceeds the human genome's size and is considered to act as a virtual organ that participates in host physiological functioning (Wang and Kasper, 2014). Gut microbes play a role in human physiology through several mechanisms including their contribution to nutrient and xenobiotic metabolism (e.g., synthesis of vitamins, digestion of oligo, and polysaccharides, drugs, etc.) and to the regulation of immune and neurodendocrine functions (Bäckhed et al., 2005). Some of these effects are mediated by products of bacterial metabolism, such as short-chain fatty acids (SCFA), including propionate, butyrate or acetate, which influence the gut barrier, the inflammatory tone and the metabolic homeostatic control in different tissues (Topping and Clifton, 2001).

A large number of cross-sectional studies report that alterations in the intestinal microbiota (termed dysbiosis) are associated not only with diseases affecting the intestine like inflammatory bowel disease (IBD) but also with extra-intestinal organs and systems. These include, but are not restricted to metabolic diseases [e.g., type-2 diabetes (T2D) and obesity], autoimmune arthritis, and psychiatric disorders (Cenit et al., 2017; Singh et al., 2017). Although it is still unclear whether the general features of a healthy microbiota can be defined at population level, disease seems to be accompanied by shifts in an individual's normal microbiota toward a dysbiotic composition which could aggravate disease pathogenesis, creating a vicious circle, though this is not completely proven. In physiological conditions, by contrast, the gut microbiota co-exist in mutualistic symbiosis with the host, contributing to the body's homeostatic control, through the regulation of immune, endocrine and neural pathways and functions (Romani-Perez et al., 2017). Obesity is a multifactorial condition that depends on intrinsic individual factors as well as on environmental variables. However, the dramatic increase in obesity over the last 40 years is considered to be a consequence of lifestyle changes such as a sedentary lifestyle and high-fat and high-carb/sugar diets. Furthermore, unhealthy dietary habits have been linked to alterations in the intestinal microbiota that could also contribute to the pathophysiology underlying obesity and its metabolic and psychological complications (Agusti et al., 2017; Portune et al., 2017). In this review, we focus on current knowledge of the role of the microbiota in regulating the gut-brain axis and its influence on mood and cognitive impairments linked to obesity. We also analyze the mechanisms through which the microbiota can alter the gut-brain communication, emphasizing effects on the hypothalamic pituitary adrenal (HPA)-axis, immune system and neurotransmission.

## The gut-brain axis

**Figure 1:**
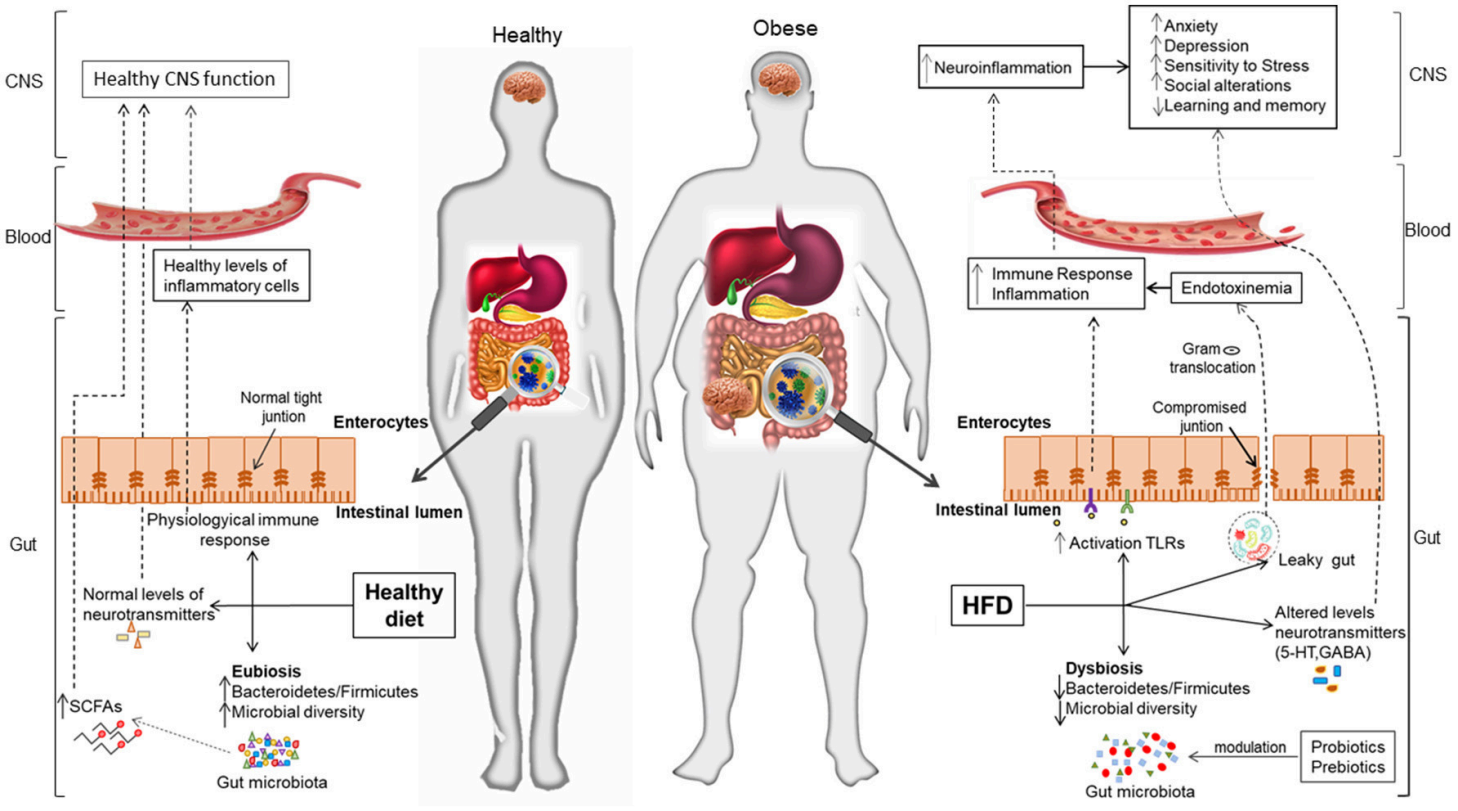
Interplay between the microbiota and the gut-brain axis in obesity and associated mental disorders. Gut microbiota contributes to regulating the gut-brain axis and maintaining health, while its alteration (dysbiosis) due to lifestyle factors (unhealthy diets, stress) is related to obesity and its adverse consequences on mood and cognition. A healthy dietary pattern (e.g., rich in fibers, vegetables, etc.) is thought to increase gut microbiota diversity and, thereby, contribute to epithelial gut integrity, immune homeostasis and normal CNS function through the gut-brain axis. On the contrary, Western-dietary patterns (rich in simple sugars and saturated fat) seem to reduce microbial diversity, promote inflammation and contribute to the *leaky gut* syndrome; this facilitates the translocation of components of Gram-negative bacteria, which increases the peripheral inflammatory tone and produces neuroinflammation and alterations in the CNS. The use of dietary strategies (e.g., probiotics, healthier diets rich in fiber, prebiotics, etc.) could beneficially impact on obesity and mental complications, via restoration of a healthy microbiota and its regulatory role in the gut-brain axis.

The gut-brain axis is a complex bidirectional communication system (see Figure 1), mediated by hormonal, immunological and neural signals, between the gut and the brain (Rhee et al., 2009). This is also a route through which the gut microbiota may impact on neurodevelopmental processes and brain functions. Dysregulation of the gut-brain axis communication is associated with metabolic diseases (Daly et al., 2011; de Lartigue et al., 2011; Grasset et al., 2017) and psychiatric and comorbid non-psychiatric disorders (Maes et al., 2007, 2008; Cryan and O'Mahony, 2011; Grenham et al., 2011; O'Mahony et al., 2011). In turn, these disorders are also frequently associated with alterations in the gut microbiota composition or function, which could also contribute to disruption of the molecular dialogue existing within the gut and brain.

The gut-brain axis is formed by the central nervous system (CNS), the enteric innervation that includes extrinsic fibers of the autonomous nervous system (ANS) and intrinsic neurons of the enteric nervous system (ENS), the HPA-axis and the intestinal microbiota. The extrinsic innervations of the GI tract connect the gut with the brain through vagal and spinal fibers, while the brain sends efferent sympathetic and parasympathetic fibers to the GI tract (Grenham et al., 2011; Browning and Travagli, 2014; Foster et al., 2017). The HPA-axis is part of the limbic system and the main regulator of the stress response. Also, the HPA-axis regulates different body processes including bowel function during digestion. Corticotrophin-releasing factor (CRF) released by the HPA-axis and different members of its family (e.g., CRF, urocortin 1, urocortin 2, and urocortin 3) are known to affect gastrointestinal tract function: bowel motility (Kihara et al., 2001; Czimmer et al., 2006), bowel permeability (Söderholm et al., 2002; Zheng et al., 2013) and bowel inflammation (Dinan et al., 2006; Gill et al., 2006). Other body processes regulated by the HPA-axis are immune functions, emotions and mood (Tsigos and Chrousos, 2002). This is supported by different studies demonstrating that activation of the stress response via the HPA-axis leads to the secretion of glucocorticoids (GCs), which in turn modulate immunity (Baschant and Tuckermann, 2010; Zen et al., 2011), as well as by studies showing that mood disorders are commonly associated with dysregulation of the HPA-axis (Nemeroff et al., 1984; Rubin et al., 1996; Deuschle et al., 1997). Stress is also linked to gastrointestinal diseases like IBD or colitis (Reber, 2012). There are several mechanisms by which gut microbiota may contribute to regulating the communication and function of this axis, including the ability to modulate immune mediators (e.g., cytokines and chemokines) and vagal nerve signaling and to generate or regulate the synthesis of neuroactive metabolites and endocrine secretions (e.g., glucocorticoids, neuropeptides, etc.) or their receptors (Moloney et al., 2014). For example, Bravo et al. (2011) observed that the administration of *L. rhamnosus* JB-1 altered mRNA GABA receptors in different brain regions in association with attenuation of stress, anxiety and depressive-like behaviors in normal healthy animals. They identified the vagus nerve as a constitutive modulator of the communication pathway between gut microbiota and brain when vagotomized animals could not show neurochemical and behavioral effects. Desbonnet et al. (2008) treated Sprague-Dawley rats for 14 days with a strain of *B. infantis*. The administration of this bacterium to *naive* rats significantly attenuated interferon (IFN)-γ, tumor necrosis factor (TNF)-α and interleukin (IL)-6 secretion following mitogen stimulation of whole blood immune cells. They also observed a marked increase in plasma concentrations of tryptophan and kynurenic acid, as well as a reduction in 5-HIAA concentration in the frontal cortex and a decrease in DOPAC in the amygdaloid cortex in the *Bifidobacterium*-treated rats when compared to controls. These results indicate that gut microbiota modulation could produce changes in immune, neuroendocrine and monoaminergic activity.

### The enteric innervation

The functionality of the GI tract (gut motility, secretory function, fluid fluxes or blood flow) is regulated by the integrated action of both CNS and the ENS. While the CNS controls gut functions through the extrinsic innervations of the ANS, the ENS may act autonomously and independently of the brain. The extrinsic innervations consist of vagal (first order cell bodies in nodose and jugular ganglia) and spinal (first order bodies in thoracolumbar and lumbosacral dorsal root ganglia) afferents which project centrally to the brainstem or spinal cord for transmitting sensory information from the gut to the CNS (Brierley and Linden, 2014). In return, the CNS sends both sympathetic efferents, which mainly induce inhibitory effects on GI tract and parasympathetic efferents that exert both inhibitory and excitatory actions (Browning and Travagli, 2014). Although the ENS receives extrinsic efferent fiber endings, it is also capable of acting as an independent nervous system. In fact, it is composed by millions of neurons including intrinsic primary afferent neurons (IPANs), which are sensory neurons, interneurons and motor neurons contained in the myenteric and submucosal plexus (Wood et al., 1999). These different neuronal populations work together to regulate several aspects of GI function (Furness et al., 2014).

The enteric innervations are separated from the intestinal luminal content, including the microbiota, by the epithelial cell barrier, the mucous layer and ion and fluid secretions (Saulnier et al., 2013). Nevertheless, study models demonstrated that the gut microbiota communicates with the enteric innervations via several possible routes that may involve intermediate interactions with immune cells and enteroendocrine cells (EECs) (Browning and Travagli, 2014). For example, EECs produce gut hormones (e.g., cholecystokinin (CCK) or glucagon-like peptide 1 (GLP-1) in response to bacterial stimuli, which also modulate enteric innervation activity; in turn, enteric neurons synapse onto EECs allowing mutual feed-back. Therefore, the microbiota could influence the ENS through its direct interaction with EEC functioning. Similarly, the ENS could sense immune signals primarily triggered by gut microbiota-immune interactions or sense the microbial molecular environment and then activate an immune response that could modify the microbiota.

Although we have limited understanding of how the intestinal microbiota influences the gut-brain axis through neural pathways and, therefore, the CNS, different study models have confirmed such interactions. The most direct evidence of the gut microbiota's role in regulating the nervous systems comes from comparing GF animals to conventionally colonized ones. Dupont et al. (1965) demonstrated that the architecture and size of the myenteric plexus were abnormal in GF rats. Anitha et al. (2012) showed that in the myenteric plexus of the colon and in the distal ileum there was a decrease in the number of nitrergic neurons in 4-week-old GF mice. The vast literature comparing GF animal models to colonized models also provides evidence that the microbiota can influence ENS development (Hyland and Cryan, 2016). Furthermore, the gut microbiota is also involved in gut barrier maintenance, since GF animals present a slower turnover of epithelial cells (Abrams et al., 1963), which could also impact on the translocation of molecules to the lamina propria and the blood stream, and the signaling process from the gut to the brain and its function.

In line with these findings, recognition of the intestinal microbiota by Toll-Like Receptors (TLR's) is essential to promote epithelial cell proliferation and regulate innate immunity (Rakoff-Nahoum et al., 2004). TLRs are also expressed by enteric neurons in the GI tract and, therefore, constitute signaling molecules that could mediate the cross-talk between the microbiota and the ENS (Koppel and Balskus, 2016). TLR2 and TLR4 play an important role in enteric innervations and small intestinal function. Cario et al. (2004) showed that redistribution of the tight junction protein zonula occludens-1 (ZO-1) was directly elevated by TLR2 activation, suggesting that TLR2 may enhance epithelial integrity. TLR2 is also involved in regulating GI physiology and enteric neurochemistry, since TLR2 knock-out mice present a reduction in the distal ileal neuron number, glial cells and myenteric ganglion area, as well as structural abnormalities in the submucosal plexus (Brun et al., 2013). Similar alterations were observed in TLR4 knock-out mice (Brun et al., 2013), showing a reduction in *in vivo* transit coupled with important changes in neurochemistry (Anitha et al., 2012). Recently, TLR2 has been shown to play an important role in the serotonergic system in the small intestine. Latorre et al. (2016) showed that TLR2 activation inhibits the serotonin transporter (SERT), thereby increasing serotonin (5-HT) contents in the small intestine. Dysregulation in the serotonergic system has been related to chronic inflammatory diseases such as intestinal bowel diseases (IBD) (Mawe and Hoffman, 2013) or diarrhea (Spiller, 2008). Besides, TLR2 can be activated by dietary saturated fatty acids (Hwang et al., 2016) and by HFD-induced intestinal dysbiosis, leading to overgrowth of potential pathogens like lipopolysaccharides (LPS)-producing *Proteobacteria*, as reported in our previous study (Moya-Perez et al., 2015; Agusti et al., 2017).

### Central nervous system (CNS)

The communication between CNS and gut is mediated by secretion of signaling molecules by neurons, immune cells and enterochromaffin cells (ECs), all of which are regulated by the brain and strongly influence the gut microbiota (Carabotti et al., 2015). The CNS, consisting of the brain and spinal cord, is responsible for integrating and coordinating all bodily information. As explained previously, there is a bidirectional transmission of information from the gut to the CNS, and from the CNS to the gut, via afferent and efferent neural, endocrine, and immunological signals between the CNS and the GI system (Romijn et al., 2008). This communication is classically known to regulate energy balance through satiety signals, among other factors (Wang and Kasper, 2014).

#### Central regulation of the energy balance

The CNS integrates environment and internal signals with information about energy needs and availability to produce a behavioral response including satiation signals, which induce fullness signals to stop eating (Woods and D'Alessio, 2008). Many of these satiation signals are mediated by peptides produced by EECs from the wall of the GI tract and transported from the blood to the brain, although some of these peptides are also produced in the CNS (Wang and Kasper, 2014). Many of the satiety signals are anorexigenic hormones such as peptide YY (PYY), GLP-1, gastric inhibitory neuropepetide (GIP), CCK, oxyntomodulin (OXM), and prouroguanylin (Pimentel et al., 2012). These signals together with those of neuropeptides, such as pro-opiomelanocortin (POMC) and cocaine and amphetamine regulated transcript (CART), are activated during the postprandial period. However, during the fasting period, orexigenic hormones like ghrelin are released mainly in the stomach and excreted to the peripheral circulation (Müller et al., 2015). Also, different neuropeptides like agouti-related protein (AgRP) and neuropeptide Y (NPY) are activated in hypothalamic neurons during fasting to produce a feeling of hunger and a behavioral response: i.e., to start eating (Schwartz et al., 2000; Leibowitz and Wortley, 2004; Valentino et al., 2011). Therefore, changes in ingestive behavior in response to CNS appetite control could influence the nutrient availability for the gut microbiota and subsequently their composition. In turn, modulation of gut microbiota and its activity by dietary intervention can also modify satiety signals. When gut bacteria metabolize prebiotics like oligofructose (OFS) or inulin, they produce SCFAs that can increase the gene expression of GLP-1 (Delzenne et al., 2005) and PYY (Karaki et al., 2006) in the intestinal tract inducing satiety. Of the SCFAs, butyrate acts as the main energy source for colonic cells and strengthens the gut barrier function and exerts anti-inflammatory effects (Andoh et al., 2003). Sodium butyrate administration also exerts an antidepressant effect related to the increased expression of brain-derived-neurotrophic factor (BDNF), which is diminished in mood disorders (Wei et al., 2015). Therefore, SCFAs generated by the microbial metabolic activity in the intestine are demonstrated to exert potential beneficial effects acting via the gut-brain axis in different study models. Bile acids also show a role in the regulation of the metabolic pathway through its binding to specific receptors like Farnesoid X receptor (FXR) involved in cholesterol production, glucose metabolism and bile acid synthesis or TGR5 (G-protein coupled receptor specific for bile acids) involved in energy expenditure in brown adipose tissue, obesity prevention and insulin resistance. Besides, microbiota is involved in the synthesis of bile acids and converts primary bile acid in secondary bile acids. This is evident in GF animals that show low bile acid diversity than its controls (Greiner and Backhed, 2011; Tomkin and Owens, 2016).

#### Central regulation of the peripheral immune system

The CNS can regulate the transcription of peripheral immune response genes (Dantzer et al., 2008) via HPA-axis or via sympathetic nervous system (SNS) (McEwen, 2007). This mechanism enables the CNS to modulate the activity of internal physiological processes to optimally adapt to external conditions, like a threat in the environment. In this situation the HPA-axis produces GCs, which in the periphery modify metabolic and developmental processes, and suppress the pro-inflammatory and antiviral immune response in the short-term (Besedovsky et al., 1986; Berkenbosch et al., 1987; Sapolsky et al., 1987; Rhen and Cidlowski, 2005). The SNS uses another neural pathway that enables the CNS to modulate the innate immune system through the nerve fibers that release noradrenaline (NA) into the primary and secondary lymphoid organs, involved in haematopoiesis and interactions between antigen-presenting cells and lymphocytes (Nance and Sanders, 2007). The stored adrenaline can be released to the systemic circulation from the adrenal glandule stimulated by the nerve fibers from the SNS, suppressing type I interferon-mediated antiviral response (Collado-Hidalgo et al., 2006) and upregulating the transcription of pro-inflammatory cytokines (Cole et al., 2007).

Neural and neuroendocrine signals like serotonin, dopamine or cytokines can also be secreted into the gut lumen by neurons, immune cells and ECs. For example, an intrathecal injection of TRP (analog of thyrotropin-releasing hormone) into the cerebrospinal fluid (central injection), which regulates the stress response to cold temperatures, produces a stomach lumen secretion of serotonin, possibly mediated by vagal activation of gastric ECs (Stephens and Tache, 1989; Yang et al., 1992). Another example is the secretion of tryptase and histamine (Mast-cell products) into the human jejunum in response to stress produced by cold pain. Other mast-cell products could be secreted into the gut lumen, including serotonin and corticotrophin-release hormone (CRH) (Santos et al., 1998).

#### Immune modulation of the CNS

Some studies that have associated gut infections with CNS alterations. For example, chronic infection with *Helicobacter pylori* produces anxiety-like behaviors in mice, induces structural and functional changes in the ENS, as well as changes in feeding patterns increasing the frequency and decreasing the quantity of food consumed. The latter are associated with a decrease in POMC expression in arcuate nucleus and increases of TNF-α in median eminence (Bercik and Collins, 2014). *Campylobacter jejuni* infection also produces anxiety-like behaviors without increasing inflammatory markers. In the latter case, the gut-brain communication might be mediated through activation of vagal ascending pathways (Goehler et al., 2008). Chronic infection with *Trichuris muris* also induces anxiety-like behavior in mice, decreased BDNF expression in the hippocampus, accompanied by a mild increase of TNF-α and IFN-γ as well as kynurenine in plasma. Administration of *Bifidobacterium longum* restored the behavioral alterations and BDNF but not the cytokines and kynurenine, proving that intervention in the gut modifies behavior (Bercik et al., 2010).

Gut pathogens, such as enterohaemorrhagic *E. coli* O157:H7, have been shown to have binding sites for enteric neurotransmitters, NA and adrenaline (ADE), on their cell surface, whose binding activates their pathogenic features and the use of adrenergic antagonists blocked this activation (Hughes and Sperandio, 2008). This suggests that the communication between enteric neurotransmitters (produced by the host) and the gut microbiota could be involved in gut infections and the inflammation associated to this process.

#### Impact of gut microbiota on CNS: role of neurotransmitters

Brain is the main modulator of gut homeostasis, controlling the motility, secretion of acid, bicarbonates and mucus (Carabotti et al., 2015). Studies have investigated how microbiota impacts the CNS, both, physiologically and pathologically (see Figure 1). The impact of gut microbiota on the CNS and behavior has been demonstrated in intervention studies with probiotics or antibiotics in rodents and, in a few cases, in humans. For example, some probiotic strains belonging to the genera *Bifidobacterium* and *Lactobacillus* have been reported to improve mood and reduce anxiety symptoms in patients with IBD and chronic fatigue syndrome (Logan and Katzman, 2005; Shadnoush et al., 2013). Also, intestinal dysbiosis has been linked to intestinal and systemic inflammatory tone, which is a mechanism contributing to mood disorders such as depression (Dowlati et al., 2010) in inflammatory conditions such as Irritable Bowel Syndrome (IBS) (Dai et al., 2013) and presumably in obesity. The mechanisms by which probiotics could mediate these effects include their possible immune regulatory properties explained above and their ability to modify neurotransmission. 5-HT is a monoamine neurotransmitter produced from tryptophan, ingested with the diet. This neurotransmitter is well known for its role in cognition and mood in the brain. However, 95% of 5-HT is produced in the gut, specifically by ECs of the mucosa and in the nerve terminals of ENS neurons. The classical functions assigned to 5-HT in the GI tract are related to its participation in GI motility, secretion and pain perception. 5-HT also has neuroprotective, trophic factor actions and pro-inflammatory actions in the gut (Mawe and Hoffman, 2013; Moloney et al., 2014). The regulation of mood and cognition depends on the availability of tryptophan on the CNS, which in turn depends on the availability of peripheral tryptophan, which is altered in GF mice. Clarke et al. (2013) demonstrated that the absence of gut microbiota in GF mice in early-life increased plasma tryptophan, suggesting a possible humoral pathway by which the microbiota could influence CNS serotonergic neurotransmission. Also, they observed an increase in 5-HT and its main metabolite, hydroxyindoleacetic acid, in hippocampus compared to normal animals. Furthermore, the colonization of the GF animals post weaning was not sufficient to reverse the CNS neurochemical consequences in adulthood due to a lack of microbiota in early life, even though the baseline values of tryptophan availability were restored. Nishino et al. (2013) also showed that GF mice exposed during 24 h to the SPF environment developed a normal (SPF) microbiota and that was accompanied by a decrease in the anxious-like behavior and an increase in ADE,DA and 5-HT brain levels. Other authors showed that GF animals present a significant reduction of 5-HT in serum compared to normal mice (specific pathogen-free, SPF) (Yano et al., 2015). Yano et al. (2015) also reported that indigenous spore-forming bacteria (Sp) from the mouse and human microbiota promote 5-HT biosynthesis from colonic ECs. These authors also showed that increased luminal concentration of specific microbial metabolites elevated colonic and blood 5-HT in GF mice. This study showed that host-microbiota interactions are very important in regulating essential 5-HT-related biological processes. Agusti et al. (2017) showed that 5-HT is significantly decreased in hippocampus of HFD-fed mice and this correlated with anhedonic-depressive-like behavior. The administration of *B. pseudocatenulatum* CECT 7765 attenuated the obesity associated depressive-like behavior and significantly increased the 5-HT concentration in hippocampus by modifying the gut microbiota. The use of probiotics in animal models of depression has shown some efficacy. For example, a strain of *Bifidobacterium infantis* restored the forced swimming test (FST) and decreased pro-inflammatory cytokines regulating tryptophan metabolism and the neurotransmitters in the CNS in a rat model of depression induced by maternal separation (Desbonnet et al., 2008, 2010).

GABA can also be affected by the gut microbiota. Bravo et al. (2011), as we explain briefly in section 2, observed that the administration of *L. rhamnosus* JB-1 altered mRNA GABA receptors in different brain regions of mice. GABA b1b receptor expression was increased in cortical cingulate and prelimbic regions, whereas it decreased in the hippocampus, amygdala, and locus coeruleus. In the prefrontal cortex (PFCx) and amygdala there was a decrease in GABA Aα2 mRNA, but an increase in the hippocampus. This was accompanied by attenuation of stress, anxiety and depressive-like behaviors in healthy animals. Results of the same study confirmed that communication between the microbiota and the brain occurred partly via the vagus nerve, which provided information from the lumen to the CNS. This was evident when vagotomized animals failed to show neurochemical and behavioral effects. Thus, the vagus nerve was identified as a constitutive modulator of the communication pathway between the gut microbiota and the brain. Other authors have also shown that components of the microbiota are able to produce molecules that act as local neurotransmitters in the ENS, for example GABA, 5-HT, acetylcholine, melatonin or histamine (Iyer et al., 2004; Portune et al., 2016).

### The HPA-axis

Gut microbiota is thought to play a role in mechanisms governing the stress response via the HPA-axis, whose deregulation has also been related to obesity. This connection was first evidence in Cushing syndrome patients who present high levels of cortisol, glucose intolerance, hypertension and upper body obesity (Bjorntorp and Rosmond, 2000; Nieuwenhuizen and Rutters, 2008).

The interconnection between the gut microbiota and the stress response via the HPA-axis was initially evidenced in GF and intentionally colonized rodents. In Sudo et al. (2004) showed that the response to stress via the HPA-axis was exacerbated in GF animals compared with SPF animals and it was accompanied by an increase in BDNF expression in cortex and hippocampus. The exaggerated response to stress was reversed by gut colonization with a strain of *B. infantis* and partially reversed with SPF stools at an early stage, but not later in life. This finding indicates that exposure to microbes at an early developmental stage is required for the HPA system to become completely susceptible to inhibitory neural regulation. O'Mahony et al. (2009) showed that early life stress induced by maternal separation also produces changes in fecal microbiota, supporting the idea that stress in early stages alters gut microbiota. On the other hand, modification of gut microbiota by a probiotic could also modulate the HPA-stress response. For example, Ait-Belgnaoui et al. (2012) demonstrate that the oral administration of *L. Farciminis* suppressed stress-induced hyperpermeability, endotoxemia and prevented HPA axis stress response and neuroinflammation.

As we indicate above, stress is an important factor in obesity and eating behavior which could also be interlinked with the microbiota. Numerous studies confirm that the HPA-axis plays an important role in the onset of metabolic alterations and obesity (Desbriere et al., 2006; Abraham et al., 2013; Champaneri et al., 2013). There is a positive association between stress, weight gain, adiposity and body mass index (BMI) (Torres and Nowson, 2007; Block et al., 2009), as well as with basal glucose, basal insulin and resistance to insulin (Sinha and Jastreboff, 2013). Furthermore, the connection between stress and metabolic dysfunction is stronger in individuals with higher BMI than in those with lower BMI (Sinha, 2008), suggesting that stress increases obesity risk especially in individuals with higher BMI. Some studies have shown that adrenal steroids increase glucose and insulin levels as well as high-caloric food intake (Sinha and Jastreboff, 2013). Chronic high levels of GCs and insulin boosted the increase in palatable food intake and abdominal fat deposition (Dallman et al., 2005; Warne, 2009). Therefore, stress could trigger metabolic dysfunction and modify eating behavior; besides, obese individuals are more sensitive to stress.

The role of the microbiota in the connection between stress and obesity has been demonstrated by, for example, the administration of potential probiotic bacteria to animal models of obesity. Thus, Agusti et al. (2017) demonstrated that basal corticosterone levels were significantly increased in mice with HFD-induced obesity. Obese animals also showed increased corticosterone levels in response to acute social stress, suggesting that obese mice were more susceptible to these stressful situations. Administration of *B. pseudocatenulatum* CECT 7765 reduced corticosterone levels in HDF-fed mice, indicating that an intervention primarily targeting the gut was able to reverse the anxiogenic obese profile.

### Immune mediators in the gut-brain axis

The immune system plays an important role in the gut-brain axis, considering that the GI tract contains the highest concentration of immune cells in the body. In GF animal models, immune defects have been observed at both levels: cellular and structural. One the one hand, Round and Mazmanian (2009) showed that there was a reduction in B-cell production of secretory IgA (sIgA) and a decrease in the intestinal T helper 17 (Th 17), CD4+ and CD8+ immune cells. They also found defects at the structural level with a decrease in Peyer's patches, lamina propria and isolated lymphoid follicles. On the other hand, the re-colonization of GF mice with a main commensal component of the gut microbiota, *Bacteroides fragilis*, proved efficient in restoring the immune maturation in gut associated lymphoid tissues.

The immune system also plays an important role in obesity (see Figure 1). The chronic nature of obesity produces prolonged low-grade activation (Gregor and Hotamisligil, 2011; Lumeng and Saltiel, 2011). Both the increase of the permeability of the gut barrier and the usual fat intake, that increases LPS absorption, contribute to this endotoxaemia (Delzenne et al., 2011; Torres-Fuentes et al., 2017). However, this endotoxaemia could be prevented by some gut bacteria which preserve the integrity of the gut barrier (Rooks and Garrett, 2016). Obesity is also associated with neuro-inflammation as described in other pathologies, including Alzheimer disease (AD) or depression (Whitmer et al., 2007; Mayeux and Stern, 2012). Obese patients are more prone to develop these types of pathologies and neuro-inflammation may underpin these associations (Guillemot-Legris and Muccioli, 2017).

Peripheral inflammation associated with obesity may lead to neuro-inflammation and could be due to the activation of components of innate immunity like Toll-like receptors (TLRs) and increased intestinal permeability (known as a *leaky gut*). *Leaky gut* is a loss of intestinal barrier integrity making it less able to protect the internal environment. This loss of integrity allows bacteria, toxins and other molecules to arrive to the bloodstream. In recent studies it has been demonstrated that gut compositional changes and inflammation related with *leaky gut* may contribute to the pathophysiology of several diseases like depression, chronic fatigue syndrome, obesity, Type-2 diabetes or IBS (Slyepchenko et al., 2017). Dietary saturated fatty acids (SFAs) can induce inflammatory responses in different organs like liver, pancreas, adipose tissue and muscle (Guillemot-Legris and Muccioli, 2017). SFAs are able to activate the TLRs expressed in intestinal epithelial and innate immune cells, specifically TLR2 and TLR4 (Hwang et al., 2016). TLR2 activation can mediate signaling cascades through myeloid differentiation factor-88 (MyD-88) and NF-κB, (Kim et al., 2013; Hayward and Lee, 2014) and TLR4 activation can activate NF-κB as well as activator protein 1 (AP-1), triggering cerebral inflammation by the upregulation of proinflammatory cytokines (Guillemot-Legris and Muccioli, 2017). The dysbiotic microbiota associated with obesity can also contribute to increasing the inflammatory tone via activation of TLRs and the subsequent production of inflammatory cytokines (Sanz and Moya-Perez, 2014).

In addition, several environmental factors such as diets rich in fats and poor in fibers, alcohol, stress, and intestinal dysbiosis observed in obese patients (Ley et al., 2006a; Riva et al., 2017) and animal models (Lin et al., 2014; Schneeberger et al., 2015) can alter the gut epithelial barrier, possibly as a secondary consequence of the inflammatory process (de Melo et al., 2017). Disruption of the intestinal barrier facilitates the translocation of components of mainly Gram-negative bacteria (e.g., LPS) from the lumen to the mesenteric lymph nodes (MNLs) and peripheral circulation (de Kort et al., 2011; Leonard and Maes, 2012). There, bacterial motifs activate the immune system through TLR2 and TLR4-biding, boosting the release of pro-inflammatory pathways (NF-κB and Mitogen-Activated Protein Kinases [MAPK]) and cytokines (e.g., TNF-α) (Chan and Riches, 2001; Wischmeyer, 2006) and increasing IgA and IgM responses to Gram-negative bacteria (Maes et al., 2007, 2008). Proinflammatory cytokines can also contribute to tight junction disruption leading to bacterial translocation (Slyepchenko et al., 2017). In addition, hypertrophy of adipose tissue increases pro-inflammatory signaling, creating a *vicious circle*. Similar processes may also disrupt the BBB allowing leukocyte infiltration into CNS, contributing to the development of mood disorders (de Melo et al., 2017) and producing neuroinflammation. The translocation of Gram-negative bacteria from the gut induce oxidative and nitrosative stress (O&N stress) processes that produce redox-derived DAMPs (damage-associated molecular patterns), which may activate TLR-2 and 4, leading to a vicious cycle known as the TLR2/4 radical cycle (Lucas and Maes, 2013). Both, endotoxemia (increased serum LPS), consequence of *leaky gut*, and adipose tissue-related inflammation could lead to insulin resistance and hyperglycemia (Fandriks, 2017). Peripheral insulin resistance may be essential to initiate a sequence of pathophysiological events in obesity. *Leaky gut* and consequent translocation of Gram-negative bacteria are also detected in MDD. This is reflected in increased IgA and IgM levels against Gram-negative bacteria, including *Hafnia alvei, Pseudomonas aeruginosa, Morganella morganii, Pseudomonas putida, Citrobacter koseri*, and *Klebsiella pneumoniae* (Maes et al., 2007, 2008). Furthermore, in MDD, these IgA/IgM levels directed to commensal bacteria are related with immune activation and O&NS (Maes et al., 2013).

## Effects of obesity on cognitive functions and mood

**Figure 2:**
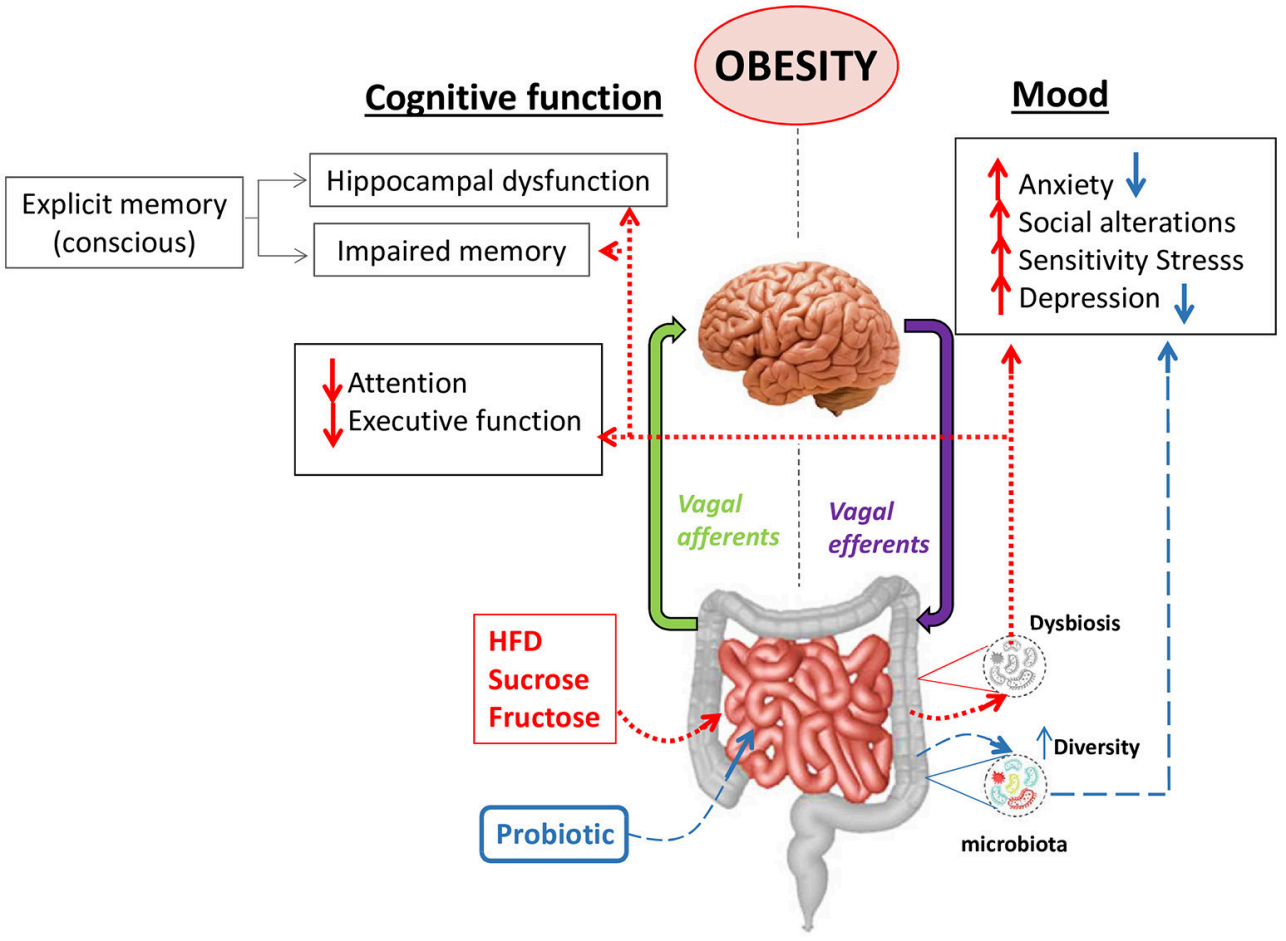
Mood and cognitive alterations in obesity: the role of the gut-brain axis. The diversity and stability of the gut microbiota can be affected by high-fat diets (HFD) or high carb diets leading to dysbiosis, which is a typical alteration observed in obesity. A dysbiotic microbiota is thought to alter the communication between the gut and the brain axis contributing to mood alterations like anxiety, depression, sensitivity to stress, social behavioral alterations and cognitive alterations like hippocampal dysfunction, impaired memory and reduction of attention or the executive function. The use of some probiotics has demonstrated to ameliorate some of the mood alterations like anxiety or depression through different mechanisms in animal models.

Obesity may contribute to cognitive damage (Johnson et al., 2016) and behavioral alterations (see Figure 2), which may partly be due to obesity-associated neuro-inflammatory processes (Guillemot-Legris and Muccioli, 2017). Obesity in childhood and adolescence could have a particularly relevant impact since these are critical periods for neurodevelopment and neuronal plasticity (Spear, 2000; Boitard et al., 2012), where negative experiences can alter brain functions, behaviors and mood states in adulthood (García-Pardo et al., 2015).

### Learning and memory in obesity

Obesity, microbiota and diet may affect episodic and semantic memory (Cheke et al., 2016; Noble et al., 2017). The explicit memory helps us to store information intentionally and consciously in order to remember past experiences. Using this type of memory we can remember autobiographic and episodic information, historical dates, vocabulary or different types of language. Various authors have demonstrated the effect of HFD or high-sugar diet on memory. For example, overfeeding in the neonatal period can lead to hippocampus damage (the main structure involved in memory tasks), causing microgliosis in this area after only 14 days of overfeeding, which can persist into adulthood (De Luca et al., 2016). In adolescents, memory impairments have been described after 4 weeks of HFD (Del Rio et al., 2016). Administration of sucrose to rats when they were progressing through puberty and adolescence may cause deficits in recognition memory tasks (Jurdak and Kanarek, 2009; Reichelt et al., 2015).

However, other data are controversial, probably due to methodological differences. Heyward et al. (2012) found that mice fed with HFD over 23 weeks exhibited intact novel object recognition but cognitive impairment in object location memory task (OLM). Also, Krishna et al. (2015) showed that novel object recognition memory was unaffected after HFD in female mice. Underwood and Thompson (2016) showed that both sexes fed HFD for 12 weeks were equally impaired in cognitive tasks.

The influence of diets rich in saturated fat on spatial learning and memory has also been reported in animal models. Collison et al. (2010) fed adult mice with a trans-fatty-acid (TFA)-enriched diet, monosodium glutamate (MSG) or a combination of both (TFA+MSG). TFA+MSG caused impairment in locating the hidden platform in the Morris Water Maze (MWM), showing a reduction in spatial cognition. Similar results were found by Guimarães et al. (2017) using the novel object recognition memory test in adult rats injected with MSG subcutaneously during the first 5 days of life to induce obesity. In adolescent animals, various authors also evidenced the relationship between juvenile HFD and spatial cognitive deficits (Boitard et al., 2012; Wang et al., 2015). Interestingly, this type of diet did not affect spatial performance in adulthood (Boitard et al., 2014) and these cognitive deficits could be reversed by exercise (Klein et al., 2016). In adult animals, caloric restriction (CR) protected CNS. Spatial memory was significantly increased in the MWM in the CR group and significantly decreased in the high-calorie (HC) group demonstrating that long-term high caloric intake induces autophagy in the hippocampus, which may increase risk to cognitive impairments (Dong et al., 2015). Mielke et al. (2006), on the other hand, found that HFD did not affect spatial memory in the MWM. Other authors have questioned whether cognitive impairment precedes obesity or is a consequence of obesity (Gurung et al., 2016). In order to test this hypothesis, obesity sensitive and resistant strain rats were fed with a standard chow diet from 4 through 20 weeks of age. At 12 weeks of age sensitive rats showed significant memory impairment on MWM compared to resistant rats. Alterations in implicit memory associated with obesity have also been investigated. Implicit or unconscious memory refers to non-intentional acts that we keep in our brain. In the rodent, additional amounts of cholesterol in the diet (0.5 % dry weight) significantly improved short-term and long-term memory (Apryatin et al., 2017). However, addition of fructose, including in combination with HFD, significantly worsened short and long-term memory in mice. Furthermore, the effects of HFD on fear conditioning may differ in female and male mice. Hwang et al. (2010) demonstrated that HFD produces more cognitive impairment in male mice than females evaluated with PAT.

Animal studies have revealed a relationship between obesity, neuro-inflammation and memory impairments. For example, Wang et al. (2016) used an anti-inflammatory agent to show that chronic rhein (the main ingredient of rhubarb plant with anti-inflammatory properties) treatment prevented HFD-induced recognition memory impairment. The rhubarb-exposed rats also had an increased bacterial diversity in the ileum, which could also contribute or reflect the beneficial effects of the treatment (Peng et al., 2014). Wang et al. (2017) demonstrated that teasaponin, an active component of tea with anti-inflammatory effects, limit unfavorable gut microbiota alterations, reduced body weight, improved glucose tolerance and prevent recognition memory impairment in HFD-fed mice; it also improved neuroinflammation, gliosis and BDNF deficits in hippocampus. Bruce-Keller et al. (2015) reported that mice receiving HFD microbiota had significant disruptions in cognitive, exploratory and stereotypical behavior compared with mice receiving control-diet microbiota in the absence of significant differences in body weight. Maternal Western diet during gestation and lactation was also shown to modify offspring's microbiota activity and cognitive responses in Yucatan pigs with modifications in hippocampal neurogenesis (Val-Laillet et al., 2017).

In humans, several studies demonstrate that obesity is associated with cognitive decline and elevated risk of neurodegenerative diseases during aging (Mazon et al., 2017). Gunstad et al. (2007) demonstrated that obese adults, with a BMI greater than 25 in an age range between 22 and 82 years old, showed a poorer executive function test performance than normal weight adults (BMI, 18.5–24.9 kg/m^2^) and some differences in the attention test performance, suggesting an association between elevated BMI and lower cognitive performance, independently of age. Several studies with obese children showed alterations in attention and attentional shifting compared to normal children (Cserjési et al., 2007; Davis and Cooper, 2011; Maayan et al., 2011; Wirt et al., 2015) and visuospatial abilities (Li et al., 2008; Jansen et al., 2011; Martin et al., 2016).

Research reports that fat and sugar intake at different life periods may also impair cognition. For example, Francis and Stevenson (2011) showed that healthy undergraduate students with high (self-reported) fat and refined sugar intake presented impaired memory tasks related to hippocampal function. Similarly, Beilharz et al. (2015) showed that hippocampal-dependent memory is sensitive to high-energy diet and they found differences between chronic and acute exposure to high-energy diets. Cournot et al. (2006) showed that higher body mass index was associated with lower cognition scores in healthy, middle-aged, non-demented participants. Another study in an Australian cohort of school-aged children, reported that higher intake of a Western diet at age 14 was associated with worse cognitive performance 3 years later (Nyaradi et al., 2014). Crichton et al. (2012) showed that the daily consumption of low-fat foods may improve cognitive performance.

SFAs have also been associated with cognitive impairments. SFA intake in young adulthood, mid and later life, increases vulnerability to cognitive deficits and even neurological diseases (Solfrizzi et al., 2011; Okereke et al., 2012). Likewise, a higher intake of carbohydrates, particularly simple sugars, has been associated with impaired cognitive functions (Roberts et al., 2012), although these associations were not detected in all studies (Halyburton et al., 2007; Brinkworth et al., 2009; Beilharz et al., 2015). Higher intake of polyunsaturated fatty acids (PUFA) and higher PUFA to SFA ratios have been associated with improved memory functions (also in children) and decreased risk of memory impairments (Kalmijn et al., 2004; Morris et al., 2004; Devore et al., 2009).

There are also some studies reporting beneficial effects of probiotics on cognitive functions in humans. For example, a cocktail of different probiotics (*Bifidobacterium bifidum* W23, *Bifidobacterium lactis* W52, *Lactobacillus acidophilus* W37, *Lactobacillus brevis* W63, *Lactobacillus casei* W56, *Lactobacillus salivarius* W24 and *L. lactis* W19 and W58) improved cognitive reactivity to sad mood in normal individuals (Steenbergen et al., 2015). A study of healthy woman treated with probiotic-fermented milk showed that probiotics were able to modulate brain activity (measured using fMRI) in brain regions involved in mediating cognitive performance (Tillisch et al., 2013).

### Anxiety and depression in obesity

Anxiety and depression are the most prevalent mental disorders in developed societies (World Health Organization, 2017). These often originate as a consequence of adverse experiences and maladaptation to stress. In addition, they show bidirectional associations with obesity and related metabolic disorders (e.g., type-2 diabetes, cardiovascular disease), as reported in both animal and human studies, summarized below.

Animal studies have investigated the effects of stress and Western diets leading to obesity on mood, and investigated the possible interactions and mediating mechanism. Santos et al. (2016) demonstrated that a high carbohydrate-containing diet administered for 12 weeks was anxiogenic and induced depressive symptoms after exposure to different stress paradigms. Chronic stress can also increase the consumption of food containing sugar and fat ingredients as a compensatory mechanism (comfort food) to reduce stress-related anxiety, which could lead to overeating and obesity long-term (Oliveira et al., 2015). In support of this hypothesis, other studies have shown that HFD administration to adult rats reduced anxiety (Leffa et al., 2015; McNeilly et al., 2015). A recent study also indicates that the combination of obesity (induced by diet) with chronic unpredictable mild stress (induced by unpredictable mild stressors like 8 h of food or water deprivation, confusing day and night, soaking the cage with water or horizontal oscillation for 20 min) induces depression and anxiety-like behaviors and the down-regulation of leptin/LepRb signaling (Yang et al., 2016). This is suggested as a possible mechanism mediating the negative consequences of obesity and stress on mood (Yang et al., 2016). In this regard, Haque et al. (2013) also indicate that leptin is important for mood and emotion regulation. They observed that animals exposed to stress (2 h immobilization) exhibited behavioral deficits, but these were reversed by exogenous leptin in a dose-dependent manner. Finger et al. (2010) showed that leptin-deficient (*ob/ob*) mice displayed higher levels of anxiety.

The connection between obesity and behavioral deficits is further supported by evidence showing that some anti-obesity therapies exert anxiolytic effects. Thus, several drugs used to treat obesity have attenuated behavioral alterations in mice, such as sibutramine (Santos et al., 2014), duloxetine (Chudasama and Bhatt, 2009), pioglitazone (Kurhe and Mahesh, 2016), celecoxib (Kurhe et al., 2014a), ondansetron (Kurhe and Mahesh, 2015), 3-methoxy-N-p-tolylquinoxalin-2-carboxamide (QCM-4) (Kurhe et al., 2014b, 2015), GPR120 agonist (Auguste et al., 2016). These findings suggest that obesity and emotional disorders, such as anxiety and depression, could share a common base. Recent works propose that part of this common base could be the gut microbiota coupled with its role in the regulation of the inflammatory tone. In support of this notion, some probiotic strains with anti-depressive effects also showed anti-obesity effects, suggesting that modulation of microbiota composition or their function could be beneficial for obesity-related depression (Schachter et al., 2017). Thus, Ohland et al. (2013) showed that administration of a strain of *Lactobacillus helveticus* restored the *Firmicutes/Bacteroidetes* ratio altered in HFD-fed mice and reduced anxiety-like behaviors. Abildgaard et al. (2017) showed that a mix of eight different strains of *Bifidobacterium* and *Lactobacillus* reduced depressive-like behaviors in HFD-fed mice in association with lowered IL-6 and TNF-α levels in serum. Therefore, the beneficial effects of some of these bacterial strains could be partly mediated by their ability to reduce the inflammatory tone affecting both obesity and mood disorders.

Epidemiological and clinical research studies in humans also support bi-directional associations between obesity, dietary patterns, and mood related disorders (see Figure 2). Systematic reviews and meta-analysis of longitudinal studies indicate that obesity increases the risk for onset of depression by 55% (de Wit et al., 2010) and depression increases the risk for obesity onset by 58% (Luppino et al., 2010). Although not all results are consistent, epidemiological data also suggest that a higher quality diet lowers the risk of onset of depressive symptoms (Molendijk et al., 2018) while consumption of high-sugar and high-saturated-fat diets is associated with greater depressive symptoms and depressed mood (Vermeulen et al., 2017). A couple of intervention studies also show that improvements in dietary quality (Mediterranean-style diet) led to improvements in depressive symptoms in adults (Opie et al., 2017; Parletta et al., 2017), further supporting the role of diet in depression. In addition, depression has been associated with alterations in the gut microbiota in a few cross-sectional studies (Cenit et al., 2017). Some of these studies also propose possible mediating mechanisms by which gut dysbiosis could contribute to mood alterations, such as changes in the tryptophan metabolism (Kelly et al., 2016).

### Social behavior and obesity

Research shows a link between obesity and social behavioral alterations in animal models but evidence in humans is very limited. In rodents, Takase et al. (2016) observed that social interactions were altered by HFD consumption in adulthood, independently of obesity, since both experimental groups, the HFD-induced obesity group and a non-obese HFD-fed group, presented social alteration. Changes in social interaction during the prepuberty period, which could cause cognitive deficits in adulthood, are modulated by diet type in rats. For example, Arcego et al. (2016) demonstrated that rats exposed to 7 days of social isolation showed memory impairment and reduced BDNF, Na+, K+, ATPase activity, MAPK, AKT and phospho-AKT levels in PFCx; however, rats exposed to this stress situation but fed with HFD showed restored memory impairment as well as the aforementioned biochemical alterations. However, Choi et al. (2017) demonstrated that sugar-sweetened beverages consumed by young mice significantly promoted social aggression in the adult life, which represents one of the most detrimental long-term outcomes of neurodevelopmental disorders (Lesch et al., 2012). Maged1-deficient mice (MAGE family genes, related with Prader-Willi syndrome (PWS), which includes hyperphagia, repetitive and compulsive behaviors, and cognitive impairment) developed progressive obesity associated with hyperphagia and a complex behavioral syndrome including reduced social interactions, memory impairments, deficient sexual behaviors, as well as increased anxiety, suggesting a connection between metabolic and behavioral outcomes (Dombret et al., 2012). They also observed a reduction in oxytocin (important hormone involved in social behavior) but not its precursor in the hypothalamus of Mage1-defficient mutants, indicating that decreased oxytocine could be responsible for the changes in social behavior.

Microbiota may also be involved in diet-related social behavioral alterations. (Buffington et al., 2016) showed a link between maternal HFD (MHFD), gut microbial imbalance, ventral tegmental area plasticity and social behavior alterations in descendants. They also observed a fewer oxytocin immunoreactive neurons in the hypothalamus of the offspring. Administration of a strain of *Lactobacillus reuteri* (reduced in microbiota of both, MHFD and their descendants) to the offspring, corrected the oxytocin levels and social deficits in MHFD progeny, suggesting that probiotics may relieve abnormalities associated with neurodevelopmental disorders. Other studies have suggested that obesity-related inflammatory processes, originating either from the adipose tissue or gut microbiota, affect the brain, leading to substantial changes in different neuro circuitry, neuroendocrine activity (impaired feedback response to cortisol), neurotransmitter metabolism and activity (alteration in basal ganglia and dopamine system), and neurogenesis (impaired in the hippocampus) (Castanon et al., 2014). The findings indicate that obesity is related to neuropsychiatric comorbidities like fatigue, anhedonia, psychomotor slowing, decreased motivation, and depressed mood (Castanon et al., 2014) as well as to social disorders like autism spectrum disorder (Gareau, 2016).

## Conclusions

The associations established between obesity and mental disorders (cognitive impairment and mood and social behavioral alterations) in epidemiological and experimental studies point to shared contributing factors and pathophysiological mechanisms. These associations could be related to dietary-induced alterations in the intestinal microbiota that, in turn, may contribute to (neuro) inflammation and dysregulation of the neuroendocrine system associated with obesity comorbid with mental impairments. This hypothesis has been confirmed to some extent in experimental models, which have investigated the effectiveness of some probiotics or compounds altering the gut microbiota, in both attenuating obesity and associated mental disorders. These studies, however, do not help us discern whether interventions affecting the gut ecosystem play a primary or secondary role in alleviating obesity-associated mental impairments. Fecal transplants have, however, provided more direct evidence for the role of dysbiotic microbiota in neurobehavioral alterations, since the colonization of lean mice with the HFD-induced microbiota of obese mice led to the neurologic complications of obesity. Although we still lack a full understanding of the mechanisms by which microbiota may influence the gut-brain axis and, thereby, brain function and behavior, studies have shed light on numerous factors, including: regulation of the gut barrier, inflammation and signaling through TLR that recognize bacterial motifs and mediate in the communication with ENS, regulation of enteroendocrine secretions and the HPA stress response, and production and regulation of host neurotransmitter levels and their receptors. However, further translational and functional studies are needed to progress in the identification of molecular targets/pathways that could be favorable modulated by microbiota-based interventions to help reduce obesity associated complications.

## Author contributions

All authors listed, have made substantial, direct and intellectual contribution to the work, and approved it for publication.

### Conflict of interest statement

The authors declare that the research was conducted in the absence of any commercial or financial relationships that could be construed as a potential conflict of interest.
